# Synergistic Effects of a Microbial Amendment and Crushed Basalt: Soil Geochemical and Microbial Responses

**DOI:** 10.1111/gcb.70705

**Published:** 2026-01-17

**Authors:** Yun‐Ya Yang, Clifton P. Bueno de Mesquita, Corey R. Lawrence, Philip D. Weyman, Daniel Dores, Tania Timmermann, Noah Fierer, Gonzalo A. Fuenzalida‐Meriz

**Affiliations:** ^1^ Andes Ag, Inc. Alameda California USA; ^2^ Department of Ecology and Evolutionary Biology University of Colorado Boulder Boulder Colorado USA; ^3^ Cooperative Institute for Research in Environmental Science, University of Colorado Boulder Boulder Colorado USA

**Keywords:** *Bacillus subtilis* strain MP1, basalt amendment, carbon dioxide removal (CDR), carbonate alkalinity, enhanced weathering (EW), metagenomics, microbial carbon dioxide mineralization (MCM), soil bacterial communities

## Abstract

Over geologic timescales, the natural weathering of silicate minerals in soils and regolith regulates atmospheric CO_2_. Although this process is slow relative to anthropogenic emissions, several strategies have been proposed to accelerate this process for climate mitigation. These include the application of finely‐ground silicate rock to increase mineral surface area (enhanced weathering, EW) and the use of microbes that catalyze mineral dissolution and CO_2_ biomineralization (microbial carbon dioxide mineralization, MCM). While both approaches show promise, their combined application has rarely been tested. Here, we examined how soil chemistry and bacterial communities respond to a basalt feedstock rich in silicate minerals, a *Bacillus subtilis* strain (MP1) previously shown to enhance weathering, and their combination. In a 91‐day soybean mesocosm experiment with slightly acidic soil (pH 6.6), MP1 persisted where applied, indicating successful inoculation via seed treatment. Basalt amendments had the strongest effect on soil bacterial community composition, whereas inoculation with MP1 exerted a smaller but detectable influence. Biogeochemical indices of weathering indicated that co‐application of basalt and MP1 enhanced carbonate alkalinity beyond basalt alone. Soil carbonate alkalinity increased with MP1 treatment both with and without basalt, while soil pH and cation exchange capacity (CEC) increased with basalt in both MP1 and non‐MP1 treatments. Total carbon was highest in the combined MP1 + basalt treatment, suggesting that MP1 may mitigate short‐term organic carbon losses associated with basalt‐driven priming. Overall, these results provide new insights into interactions between biological and mineral‐based carbon dioxide removal (CDR) strategies, suggesting that co‐application of MP1 with basalt in slightly acidic soil may enhance carbonate alkalinity while reducing organic carbon losses relative to basalt alone. Thus, pairing *B. subtilis* MP1 with enhanced weathering deployments emerges as a promising strategy to improve CDR efficiency.

## Graphical abstract

Enhancing Alkalinity and Mitigating Soil Organic Carbon Losses via the Co‐Application of *Bacillus subtilis* MP1 and Basalt. We discovered that adding the bacterium *B. subtilis* MP1 to basalt rock treatments amplifies the chemical processes needed to lock away carbon in soil. This method increases carbonate alkalinity, allowing the soil to retain significantly more total carbon compared to using rock alone. The process is both efficient and ecologically safe, as the bacteria enhance carbon capture while preserving the pre‐existing soil microbial community.

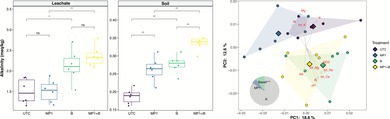

## 1 Introduction

Climate change, driven predominantly by anthropogenic greenhouse gas emissions, represents an existential threat to global ecosystems and human society. The urgency to mitigate this crisis has accelerated the development and implementation of various carbon dioxide removal (CDR) strategies aimed at reducing atmospheric CO_2_ concentrations (Caldeira et al. 2013; Amann and Hartmann 2019; Fierer and Walsh 2023; Reginato 2025). Among these approaches, soil‐based CDR technologies have garnered significant attention due to their potential for scalable deployment, durability of carbon sequestration, and co‐beneficial impacts on soil health and agricultural productivity.

Enhanced weathering (EW) is a soil‐based CDR strategy wherein the dissolution of silicate minerals, a reaction which consumes CO_2_ to produce dissolved base cations and carbonate alkalinity, is accelerated to sequester carbon with long‐term durability (Hartmann et al. 2013; Goll et al. 2021). In this study, we focus on two methods used to accelerate silicate weathering in soils. The most common method, EW, involves the application of finely ground silicate feedstocks, such as basalt, to soils, which accelerates weathering by increasing mineral surface area. An alternative approach, which we refer to as microbial carbon dioxide mineralization (MCM), leverages microbial metabolisms to enhance the dissolution of native soil silicate minerals (e.g., Timmermann et al. 2025). Microorganisms and plants are known to be important contributors to mineral weathering (Finlay et al. 2020; Vicca et al. 2022; Wild et al. 2022; Banfield et al. 1999; Verbruggen et al. 2021; Timmermann et al. 2025). The benefits realized by the MCM approach are derived from the addition of a microbe that is particularly adapted to colonize plant roots, thrive in association with them, and, as part of its metabolic processes, generate acidity that accelerates the weathering of silicate minerals.

Although these approaches share the fundamental goal of accelerating silicate mineral weathering, they differ in their technical implementation and operational scope. Feedstock additions are typically deployed in acidic soils and increases in weathering are driven by a greater abundance of weatherable minerals (Dupla et al. 2025; Deng et al. 2023). This approach requires the production, transport, and distribution of the feedstock material, which has non‐negligible financial costs and carbon emissions. Conversely, MCM uniquely integrates biological activity, via the inoculation of microbial strains into agricultural fields to catalyze weathering through processes that are only now beginning to be understood (Uroz et al. 2022; Janssens et al. 2025). The MCM approach generally requires soils with neutral or alkaline pH conditions and an abundance of native silicate minerals. Because it leverages the silicate minerals that are already present in the soil, MCM presents lower financial and carbon currency costs compared to feedstock additions.

Given the complementary nature of these approaches, a promising yet underexplored strategy is their simultaneous application. Limited studies to date have assessed the combined effect of silicate rock amendments and microbial inoculants (Niron et al. 2024; Corbett et al. 2024; Ribeiro et al. 2020). The geochemical and microbial interactions between added crushed silicate rock and inoculated weathering‐enhancing microorganisms remain poorly understood, particularly in agricultural contexts. This represents a significant limitation for optimizing these technologies and understanding their ecological impacts. Furthermore, despite growing interest in enhanced weathering, limited research has examined how the addition of crushed basalt, a widely used EW feedstock, affects pre‐existing soil microbial communities in agricultural systems (Reis et al. 2024; Chen et al. 2025). Understanding these ecological impacts is crucial for assessing the sustainability and long‐term viability of enhanced weathering deployment. Similarly, while *Bacillus subtilis* strain MP1 has been characterized for its silicate weathering‐enhancing capabilities (Timmermann et al. 2025), its persistence in soils and its potential community‐level effects on pre‐existing soil microbial communities require investigation.

We address these knowledge gaps through a controlled mesocosm experiment examining the individual and combined effects of crushed basalt addition and strain MP1 inoculation on soil geochemistry and microbial community structure. Using agricultural soil from Minnesota, USA (pH 6.6) planted with soybean, a 13‐week experiment was conducted with basalt application equivalent to 100 t per hectare with and without inoculation of MP1. Through comprehensive analysis of soil and leachate chemistry combined with metagenomic characterization of soil bacterial communities, this research provides crucial insights into the feasibility and ecological implications of combining feedstock and microbial amendments for strengthening CDR in agricultural systems.

This research contributes to the fundamental understanding of how engineered weathering enhancement strategies interact with soil ecosystems, informing the sustainable deployment of nature‐based CDR solutions at scale. To our knowledge, this study represents one of the first comprehensive investigations of soil microbial community responses to either mineralizing microbial‐ and/or rock feedstock additions and is among the few studies examining the synergistic potential of combining these approaches in agricultural soils.

## 2 Materials and Methods

### 2.1 Mesocosm Experiments

A 91‐day soil mesocosm experiment was conducted using 24 soil columns to investigate the effects of *Bacillus subtilis* strain MP1 on soil properties and microbial community structure under soybean ( *Glycine max* ) cultivation, with and without basalt amendment. The soil used for the mesocosm experiment, hereafter referred to as SBX68, was a silty clay loam (56% silt, 25% clay, and 19% sand) collected from an agricultural field in Chippewa County, Minnesota, USA. To ensure material consistency, approximately 150 kg of soil was excavated from a single pit using a backhoe. Prior to mesocosm preparation, the SBX68 soil was sieved through a 10 mm mesh to remove large stones and plant debris, and then manually homogenized. Each mesocosm (35 cm height and 10 cm diameter) was filled with 3 kg of field‐moist SBX68 soil to a total height of 30 cm, leaving 5 cm of headspace. After correcting for the moisture content of the soil, this equated to a dry mass of 2.22 kg and a soil bulk density of 0.93 g·cm^−3^ within each mesocosm.

The experimental design consisted of four treatment groups, each with six biological replicates: (i) untreated control (“UTC”), (ii) MP1 inoculated (“MP1”), (iii) Basalt‐amended (“B”), and (iv) MP1 amended with basalt (“MP1 + B”) (Figure S1). MP1 is a naturally occurring *B. subtilis* strain isolated from corn roots and rhizosphere soils. Detailed characterization of MP1 can be found in Timmermann et al. (2025). In the basalt‐amended mesocosms, crushed basalt with a grain size p80 of 125 μm was used. A mass of basalt equivalent to 10 kg·m^−2^ (100 t·ha^−1^), which falls within the range of recommended application rates (Dupla et al. 2024; Skov et al. 2024; Kelland et al. 2020), was thoroughly mixed with the SBX68 soil before filling the columns.

Each mesocosm was planted with a single soybean seed (variety Innvictis A1591XF XtendFlex). To prepare the MP1 treatment, each seed was inoculated with 1 × 10^7^ spores of MP1 suspended in 1 mL of distilled water. The concentration of MP1 spores added per mesocosm equates to 1 × 10^4^ spores per gram of soil. In the UTC treatment, 1 mL of distilled water was applied instead. The experiment was conducted in a growth chamber under the following conditions: temperature of 22°C (±5°C), relative humidity of 65% (±5%), and a 16‐h photoperiod. Plants were watered three times a week for the first 6 weeks. Detailed descriptions of the mesocosm design, sample collection, as well as the characterization of the basalt amendment, are provided in the Supporting Information.

### 2.2 Simulated Rainfall Events and Sample Collection

Beginning 6 weeks (42 days) after the onset of plant growth, six simulated rainfall events were applied to the mesocosm over a 2‐week period. The total rainfall amount was 1.75 L of deionized (DI) water per mesocosm, distributed across the six events, with each mesocosm receiving 292 mL DI water per event. The total volume of water was determined as described in Timmermann et al. (2025). The simulated rainfall schedule followed a pattern of two consecutive days of water additions, followed by 2 days without, repeating until 6 days of simulated rainfall were completed. Leachate was collected from each mesocosm during each of the six discrete rainfall events, thereby accounting for the total leachate flux throughout the experiment. Following the simulated rainfall period, plants continued to grow under controlled conditions until day 91, at which point they were harvested.

After the 91‐day experiment, all 24 mesocosms were harvested and soils were divided into three depth intervals for analyses: 0–10 cm, 10–20 cm, and 20–30 cm (Figure S1). A total of 72 soil samples (four treatments × six replicates × three depths) were collected, homogenized, and divided into two subsamples. The first was air dried, sieved to 2‐mm, and ground for soil physicochemical analyses. The second subsample was immediately stored at −80°C for subsequent metagenomic analysis. Soybean plants were harvested separately for aboveground and belowground biomass.

### 2.3 Soil Analyses

The total elemental composition of the SBX68 soil and the soil plus basalt mixture at the onset of the experiment was measured via x‐ray fluorescence (XRF) at the GeoAnalytical Laboratory at Washington State University. Soil organic matter content was determined by the loss‐on‐ignition method at 360°C (Combs and Nathan 1998) to allow for normalization of the elemental composition on a volatile‐free basis. Technical details and principles underlying these methods are described in Johnson et al. (1999) and Kelly (2018).

Soil inorganic carbon (SIC) was quantified using a gas chromatography (GC) method described in Yip et al. (2025). Soil bicarbonate (HCO_3_^−^) and carbonate (CO_3_^2−^) ions were quantified using a saturated paste extract followed by titration. Due to the complexity of this measurement, these measurements were made on a single depth‐composited sample from each column. Briefly, 200 g of dried, sieved soil was gradually mixed with deionized water to create a saturated soil paste, stirred until a smooth, glistening consistency was achieved, and then set for 1 h (Richards 1954). The paste was transferred to a Buchner funnel lined with Grade 42 Whatman filter paper, and the soil saturation extract was collected under vacuum using a WOB‐L 2546 pump (Welch, IL, USA). Titration was performed on 10 mL of extract using 0.0125 M H_2_SO_4_ delivered with a digital burette (BrandTech Titrette Bottle‐top Burettes, BrandTech Scientific Inc., CT, USA). Phenolphthalein was used to determine carbonate ions (endpoint at pH 8.3), and methyl orange was used to determine total alkalinity (endpoint at pH 4.5), following Richards (1954). In this study, “carbonate alkalinity” is defined as the combined concentration of HCO_3_^−^ and CO_3_^2−^ ions in the soil.

Additional soil physicochemical parameters were analyzed by an external commercial laboratory (Agvise Laboratories, ND). Soil pH was measured using the standard 1:1 soil‐to‐water ratio method with a calibrated pH meter (Peters et al. 2012). Exchangeable cations (Ca^2+^, Mg^2+^, K^+^, Na^+^) were extracted with 1 M ammonium acetate (NH_4_OAc, pH 7) and analyzed by inductively coupled plasma atomic emission spectrometry (ICP‐AES; Perkin Elmer Optima 5300, Optima 7300) (Warnacke and Brown 2015). Iron was extracted using DTPA‐TEA solution (diethylenetriaminepentaacetic acid—triethanolamine; 0.005 M DTPA, 0.01 M CaCl_2_, and 0.1 M TEA; pH adjusted to 7.3). A 1:2 soil‐to‐solution ratio was used, and the mixture was shaken at 180 rpm for 2 h before filtration through Whatman No. 42 filter paper (Whitney 2015). Total available Al, Ca, Mg, Na, and K were extracted by digesting 0.25 g of soil with concentrated HNO_3_, 30% H_2_O_2_, and concentrated HCl, following the EPA method (EPA Method 3050B, EPA, U.S. 1996) and concentrations were quantified by ICP‐AES (Perkin Elmer Optima 5300, Optima 7300, and Avio 500 Max series). This procedure dissolves “environmentally available” components such as carbonate, phosphate, and sulfate minerals, while leaving resistant silicates intact. Soil total carbon was quantified via dry combustion using a vario MACRO cube elemental analyzer (Elementar Americas Inc., NY), while total organic carbon was calculated by subtracting total inorganic carbon from the total carbon content (Nelson and Sommers 1996).

### 2.4 Leachate Analyses

Leachate samples, collected from mesocosms following each simulated rainfall event, were analyzed for several biogeochemical parameters. Leachate pH was measured with a benchtop probe following a multipoint calibration. Bicarbonate and CO_3_^2−^ ions were quantified via titration with 0.0125 M H_2_SO_4_ with phenolphthalein and methyl orange indicators (Richards 1954). Dissolved inorganic carbon (DIC) in leachate was measured on 4 mL of sample following the GC method described in the previous section. Remaining leachate samples were stored frozen and submitted to Agvise Laboratories (ND, USA) for analysis of concentrations of soluble cations Ca^2+^, Mg^2+^, K^+^, and Na^+^ (American Public Health Association 1998) as well as major anions including NO_3_^−^, SO_4_^2−^, and Cl^−^. Soil leachate data was assessed for charge balance. Positive charge offsets were assumed to result from sample re‐equilibration during the collection phase and were corrected by adjusting total carbonate alkalinity (HCO_3_^−^ + CO_3_^2−^) by the corresponding magnitude of the offset (Tosca and Tutolo 2023).

### 2.5 Microbial Analyses via Metagenomic Sequencing

DNA was extracted from 0.25 g of soil with a Qiagen PowerSoil Pro (Qiagen, Hilden, Germany) kit following the manufacturer's instructions. DNA was sequenced on four lanes of an Illumina NovaSeq 6000 (150‐bp paired reads), with reads across all four lanes combined by sample. Illumina adapter sequences and barcodes were trimmed with cutadapt (Martin 2011). Then, Trimmomatic (Bolger et al. 2014) was used to validate paired‐end reads and quality filter to a minimum mean quality score of 20. Sequencing depth of trimmed and filtered reads per sample ranged from 45 to 87 million reads (mean 66 million). Two samples were removed due to low sequencing depth, leaving 70 samples for downstream analyses. For taxonomic analyses, we profiled the metagenomic reads using mTAGs (Salazar et al. 2021), which identifies 16S rRNA genes and assigns taxonomy of operational taxonomic units (OTUs) against the SILVA 138 reference database (Quast et al. 2013). OTUs assigned to chloroplast or mitochondria or OTUs that were detected in two or fewer samples were filtered out with the *mctoolsr* R package (Leff 2022). 16S rRNA gene abundances from mTAGs were rarefied to 9299 sequences per sample to capture the diversity in each sample.

To determine if MP1 was present in the metagenomes, we ran Sylph (Shaw and Yu 2025) in profile mode using the MP1 genome and dereplicated GTDB r220 genomes (*n* = 113,104) as input genomes. Sylph uses k‐mer sketching and zero‐inflated Poisson statistics for effective containment average nucleotide identity (ANI) calculations even at low abundances. FastANI (Jain et al. 2018) was used to compute whole‐genome ANI between MP1 and other detected *Bacillus* strains in the soil samples.

We also conducted analyses of DNA extracted from pure basalt to determine what exogenous microorganisms may have been added to the soil from the basalt amendment. DNA from four replicates of pure basalt was extracted by vortexing 1 g of basalt with 1 mL of phosphate‐buffered saline solution. The Qiagen PowerSoil Pro kit was used to extract DNA from the supernatants. The 16S rRNA gene was amplified via PCR with the 515f/806br primer pair following the methods of the Earth Microbiome Project (Thompson et al. 2017), with normalization and cleaning of amplicons performed using beads. DNA was sequenced on an Oxford Nanopore Technologies (Oxford, UK) MinION sequencer with libraries prepared using the Oxford Nanopore Technologies Ligation Sequencing Kit V14 (SQK‐LSK114) protocol. Reads were demultiplexed with Dorado v0.8.2 (Oxford Nanopore Technologies 2024), primers were trimmed with cutadapt v4.9 (Martin 2011), and reads were quality filtered to a minimum Phred score of 23. DADA2 (Callahan et al. 2016) was then used to quality filter reads, infer amplicon sequence variants, remove chimeras, and assign taxonomy with the SILVA 138 taxonomic database (Quast et al. 2013).

To quantify the abundance of MP1 in the bulk soil at the end of the 91‐day experiment (and complement the metagenomic‐based analyses conducted using Sylph, as described above), quantitative PCR (qPCR) was performed. DNA samples were normalized to 5 ng·μl^−1^ using water as the diluent. qPCR reactions were performed in 96‐well format using 20 μL reaction volumes per well containing the following: 1× SYBR Green Universal Master Mix (Thermo, Product #4309155), 50 nM each primer, and 5 ng template DNA. The primers were designed to target a gene of unknown function in the MP1 genome that is absent from related *Bacillus subtilis* species. The sequence of the forward primer was 5′‐CCCCTGGCTAATCGAACATCATA‐3′, the sequence of the reverse primer was 5′‐TCGGAGACATTTGGAGCTATGC‐3′, yielding an amplicon 169 bp in length. The reactions were denatured at 95°C for 10 min followed by 40 cycles of 95°C for 30 s, 60°C for 60 s with data collection, and 72°C for 60 s. A melting curve followed that denatured at 95°C for 60 s and then began at 55°C and ramped up by degree to 95°C with data collection at each degree. qPCR was run on an MX3000P instrument (Stratagene) using software MxPro V4.10. The standard curve was created by adding MP1 cells to 250 mg soil from a UTC sample, and the samples were extracted using the Powersoil kit (Qiagen) according to the manufacturer's instructions. A linear standard curve was generated for Ct values resulting from soil samples spiked with 10^2^ to 10^6^ cells per extraction. Soil by itself and the no‐template control failed to return a Ct value consistent with there being no template to amplify.

### 2.6 Statistical Analyses

Based on the distribution and homoscedasticity of the data, either a parametric *t*‐test or a nonparametric Mann–Whitney *U* test (Wilcoxon rank‐sum) was applied to evaluate significant differences (*p* < 0.05) in soil properties among treatments. All statistical analyses of soil and water physicochemical properties were performed using the JMP software package (JMP 18.2.0, SAS Institute). To assess the statistical power of the experimental design regarding key biogeochemical indicators that showed directional but non‐significant trends, a post hoc power analysis was conducted. Specifically, we evaluated the cumulative fluxes of total alkalinity and total cations, as well as leachate alkalinity concentrations. Using the observed means and standard deviations from the basalt‐amended treatments (MP1 + B vs. B), we calculated the effect sizes (Cohen's *d*) and determined the sample size (*N*) required to achieve a statistical power of 0.80 (1 − *β*) at a significance level of *α* = 0.05. This analysis was performed to distinguish between an absence of effect and an absence of statistical evidence due to sample size constraints in short‐term mesocosm experiments.

Effects of MP1, Basalt, and their interaction on microbial taxonomic composition (Bray–Curtis dissimilarity) were tested with permutational analysis of variance (PERMANOVA) implemented in the *vegan* R package (Oksanen et al. 2022). If the interaction term was not significant, the model was rerun without the interaction term. Effect sizes were calculated as eta‐squared values based on the model sum of squares. Differences in community composition were visualized with principal coordinates analysis (PCoA). Associations between the PCoA and soil chemical variables were assessed with ‘envfit’ in *vegan* and variables with *p* < 0.05 were added as vectors onto the PCoA plot. Relationships between soil chemical variables and Bray–Curtis dissimilarity were tested with distance‐based redundancy analysis with the ‘dbrda’ function in *vegan* and forward stepwise model selection with ‘ordistep’ was performed to select the best combination of predictor variables. Differential abundance analysis of taxa was performed with ANCOM‐BC, which by default omits OTUs that are not present in either treatment from the analysis (Lin and Peddada 2020).

## 3 Results

### 3.1 Initial Geochemical Composition of Soil and Soil + Basalt Mixture

The SBX68 soil has a pH of 6.6 ± 0.03 (*n* = 3), a cation exchange capacity (CEC) of 20.5 ± 0.2 meq/100 g, soil organic carbon (SOC) content of 1.6% ± 0.03%, and SIC of 0.06% ± 0.004%. The mineral fraction of the SBX68 soil is primarily composed of quartz (50%), K‐feldspar (16%), and albite (14%) with a mixture of pyroxenes/amphiboles, anorthite and other silicates making up the balance. The basalt feedstock is composed of anorthite (63%), pyroxenes/amphiboles (28%), and quartz (9%). Amending the SBX68 soil with basalt increased the pH to 6.8 and slightly enriched the amounts of anorthite and pyroxene amphiboles, while slightly diluting concentrations of quartz, K‐feldspar, albite, and soil organic matter (Table S1). Measurements of elemental content via XRF showed small increases in the concentration of Ca, Mg, Na, and Fe‐bearing oxides (e.g., between 0.05 and 0.44 wt %) and similarly small decreases in concentrations of Si and K‐bearing oxides, compared with the unamended soil (Table S2).

### 3.2 MP1 Effects on Soil and Leachate Biogeochemistry

#### 3.2.1 pH

**FIGURE 1:**
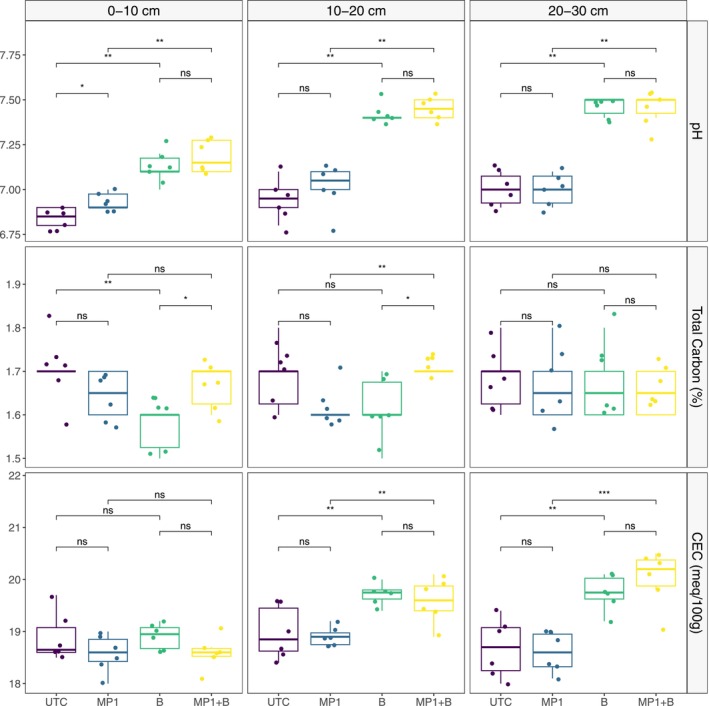
Soil pH, total carbon and cation exchange capacity (CEC) by treatment and soil depth increment. For pH and total carbon, groups are compared using the non‐parametric Mann–Whitney *U* (Wilcoxon rank‐sum) test, where: Ns, Not significant; *p < 0.05; **p < 0.01. For CEC, groups are compared using the parametric *t*‐test (unpaired), where: Ns, Not significant; **p < 0.01; ***p < 0.001. Note that *y*‐axes are scaled to the data range to clearly visualize the distribution and variability across treatments.

At the end of the 91‐day experiment, soil pH was elevated above initial values for both basalt‐amended and unamended soils. In the control and MP1 treatments, pH values in the basalt‐amended soils were significantly higher (*p* < 0.0001) than in unamended soil across all depth increments. Within each soil column, the mean soil pH was generally higher for MP1 versus UTC with significant differences at the 0–10 cm soil increment (Figure 1). This is consistent with the metagenomic results where MP1 was found in that soil depth increment (see Section 3.3 below).

#### 3.2.2 Alkalinity

**FIGURE 2:**
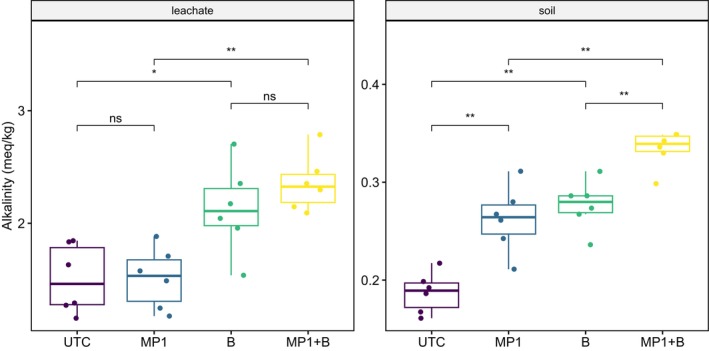
Comparison of carbonate‐alkalinity for liquid (leachate) and solid (soil) phases. Carbonate‐alkalinity in the leachate samples has been corrected for charge imbalance (see text for details). Both sets of data are reported per kg of soil in the columns. Groups are compared using the non‐parametric Mann–Whitney *U* (Wilcoxon rank‐sum) test where: Ns, Not significant; *p < 0.05; **p < 0.01. Note that y‐axes are scaled to the data range to clearly visualize the distribution and variability across treatments.

Carbonate alkalinity (CO_3_^2−^ + HCO_3_^−^) was significantly higher (*p* < 0.0001) in soil and leachate for samples from basalt‐amended soils (Figure 2). Importantly, there was an MP1 treatment effect on carbonate alkalinity measured in soil, which was significantly higher (*p* < 0.0001) in the MP1‐treated soils compared to the UTC soils, regardless of basalt amendment status (Figure 2). Leachate alkalinity was higher for MP1 versus UTC, especially in the basalt‐amended soils; however, the differences were not significant (*p* > 0.05). When normalized to the mass of soil in each column, carbonate alkalinity measured in the soil accounted for roughly 12% and 14% of the total carbonate alkalinity in the UTC and MP1 treatments, respectively.

#### 3.2.3 Carbon

**FIGURE 3:**
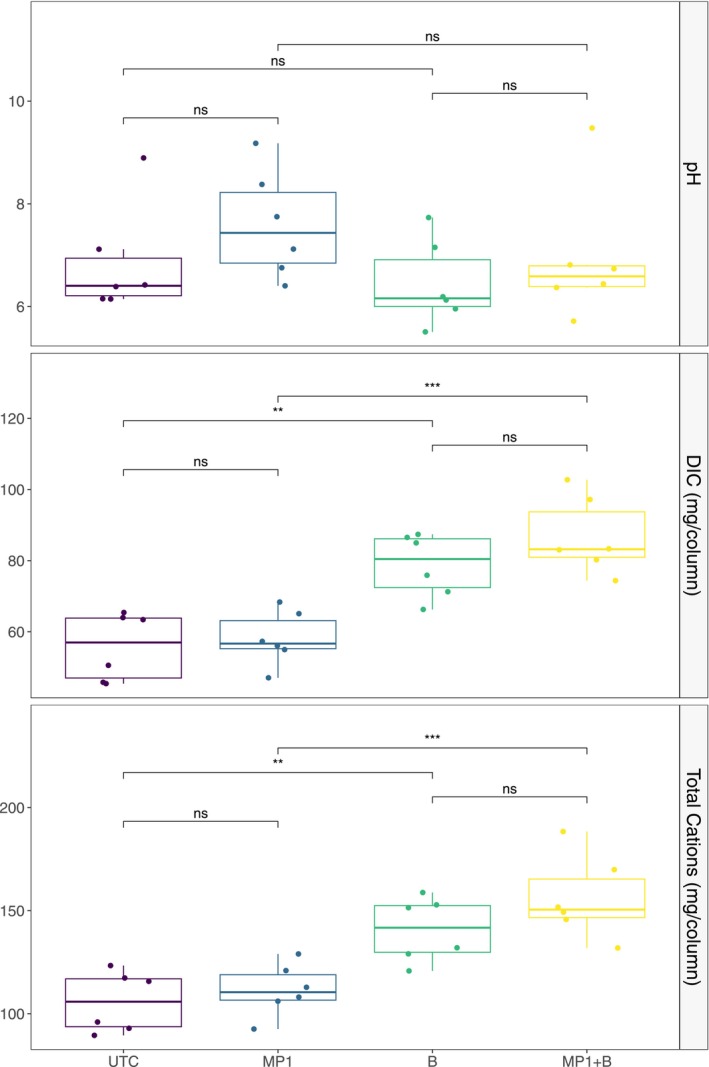
Leachate chemistry including pH averaged by column over all wetting events, and the total fluxes of dissolved inorganic carbon (DIC) and base‐cations (Ca, Mg, Na, and K). For pH groups are compared using the non‐parametric Mann–Whitney *U* (Wilcoxon rank‐sum) test where: Ns, Not significant. For DIC and total cations groups are compared using the parametric *t*‐test (unpaired), where: Ns, Not significant; **p < 0.01; ***p < 0.001. Note that *y*‐axes are scaled to the data range to clearly visualize the distribution and variability across treatments.

Soil total carbon (TC) concentrations at the end of the experiment were similar to or higher than the initial values in both basalt‐amended and unamended soils. In the basalt‐amended soils, TC concentrations were lower in UTC treatments compared to MP1 (*p* < 0.05) for the upper two depth increments (Figure 1). There were no significant differences in TC between MP1 and UTC treatments in the unamended soils. Soil inorganic carbon was near the detection limit of our method for all soils in this study (data not shown). As a result, treatment effects on soil carbon are primarily driven by differences in SOC. Cumulative DIC fluxes were significantly greater from basalt‐amended versus unamended soils, but there were no differences in DIC fluxes between the UTC and MP1 treatments (Figure 3).

#### 3.2.4 Cations and Cation Exchange Capacity

At the end of the 91‐day experiment, total available Ca (*p* < 0.001), Mg (*p* < 0.01), and Na (*p* < 0.001) concentrations were higher, and K (*p* < 0.001) was lower, in basalt‐amended versus unamended soil (data not shown). Exchangeable Ca (*p* < 0.0001) and Mg (*p* < 0.01) were also higher in the basalt‐amended soils. There were no significant differences in total available or exchangeable cation concentrations between UTC and MP1 treatments in either soil matrix. The soil‐column averaged CEC was significantly higher in the 10–20 and 20–30 cm depth increments of the basalt‐amended soils, the absolute values and the significance level increasing with depth (Figure 1). There were no significant differences in CEC between UTC and MP1 treatments when considered across the column or by individual depth increments.

The total flux of cations (calculated as leachate volume collected × concentration) was significantly higher from basalt‐amended soils (Figure 3). This difference was primarily due to significantly higher amounts of Ca and Mg export from basalt‐amended soils; export of Na was only higher in the UTC treatments and K fluxes did not differ significantly (Figure S2). Although the mean values of all cation fluxes were consistently higher from MP1 versus UTC treatments (with or without basalt), these differences were not significant (Figure S2).

#### 3.2.5 Multivariate Clustering of Geochemical Data

**FIGURE 4:**
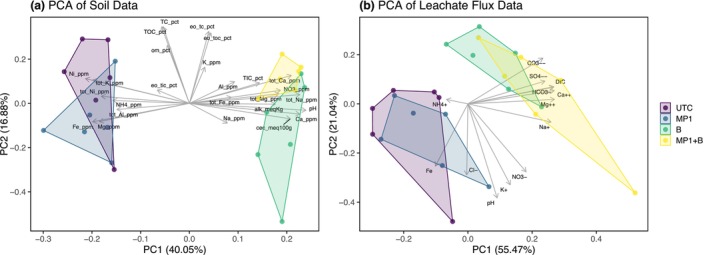
The PCA results using (a) soil column averaged concentration, and (b) soil leachate flux summed across all wetting events. Individual points represent replicate columns.

Taking into account all geochemical data, a Principal Component Analysis (PCA) indicated a clear and consistent separation of basalt‐amended versus unamended soils (Figure 4a) and leachate (Figure 4b). In both plots, these groupings separated along Principal Component 1 (PC1) and were driven by higher amounts of total available Ca, Mg, Na, and inorganic carbon in the basalt‐amended columns. Differentiation in PCA‐based clustering of soil data was similar for all depth increments considered individually (Figure S3).

### 3.3 Soil Microbial Communities

**FIGURE 5:**
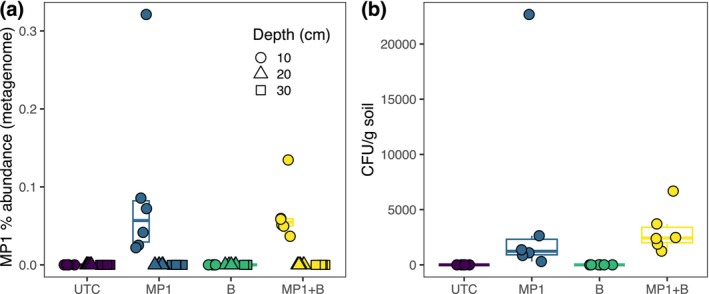
MP1 was detected in the top 10 cm of all soils to which it was added (MP1 and MP1 + B treatments) using two methods. (a) Relative abundance of the MP1 genome (relative to all detected genomes in GTDB r220) as calculated by Sylph from shotgun metagenomic reads. (b) Abundance of MP1 in the first soil depth increment (0–10 cm) as calculated by qPCR with MP1‐specific primers. Only surface soils (0–10 cm) were analyzed via qPCR.

We first used the metagenomic data to see if we could detect the presence of the MP1 strain in the treatments that received the MP1 seed inoculum to test whether the inoculum effectively colonized the soils. Based on the genome‐specific analyses (see Methods), we found that the MP1 strain was detected in the top 10 cm in all samples to which it was added (Figure 5a) with relative abundances (compared to all other detected bacterial genomes) of 0.02% to 0.32%. In contrast, the MP1 genome was not detected in any of the deeper soil layers, even in columns to which it had been added at the surface. Four other *Bacillus* genomes were detected in addition to MP1: *Bacillus altitudinus* (GCF_000691145.1), *Bacillus pumilus* (GCF_900186955.1), *Bacillus pumilus_O* (GCF_009937765.1), and *Bacillus safensis* (GCF_000691165.1). These genomes can be confidently identified as distinct from MP1 by Sylph, since average nucleotide identity between these other *Bacillus* genomes and the MP1 genome ranged from 78.16% to 79.12%.

The detection of MP1 in the top layer of the columns treated with MP1 was further confirmed and quantified with our targeted qPCR assay. In line with the metagenomic results, qPCR detected MP1 in both conditions in which it was added (MP1 and MP1 + B treatments; Figure 5b). We estimated the abundance of MP1 in the bulk soil of the top layer to be around 4800 CFU·g^−1^ for the MP1 treatment and around 3100 CFU·g^−1^ for the MP1 + B treatment. MP1 was not detected by qPCR at any depth in the other experimental treatments that did not receive the MP1 inoculum.

**FIGURE 6:**
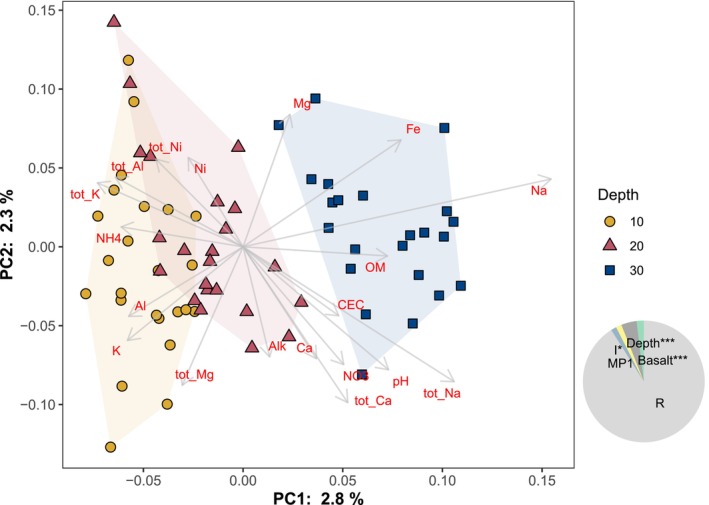
Principal coordinates analysis of Bray–Curtis dissimilarity at the OTU level, with hulls showing the clustering by depth, and vectors showing associations with soil variables (envfit, *p* < 0.05). Pie inset shows the effect sizes (eta‐sq) from ANOVA (* *p* < 0.05, ***p < 0.001).

**FIGURE 7:**
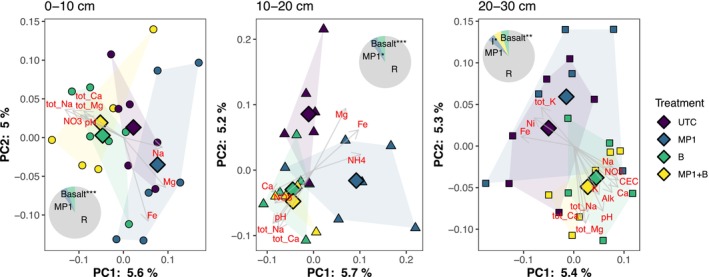
Principal coordinates analysis of Bray–Curtis dissimilarity at the OTU level across the three depths, with hulls showing the clustering by treatments, and vectors showing associations with soil variables (envfit, *p* < 0.05). Diamonds represent the treatment centroids. Pie insets show the effect sizes (eta‐sq) from ANOVA (*p < 0.05, **p < 0.01, ***p < 0.001).

Bacterial community composition primarily varied as a function of soil depth, with secondary effects of treatment (Figure 6). At the phylum level, Desulfobacterota and Chloroflexi increased in relative abundance in deeper soils, while Patescibacteria, Planctomycetota, Bdellovibrionota, Cyanobacteria, Myxococcota, Latescibacterota, and Verrucomicrobiota decreased in relative abundance (Kruskal‐Wallis, *p*_Bon_ < 0.05). Within soil depths, bacterial communities were significantly affected by the treatments, with effects of basalt addition generally outweighing effects of MP1 addition, similar to the results for soil chemical variables. In the top 10 cm, basalt addition significantly affected community composition while MP1 addition did not (Figure 7 and Figure S4), whereas at 20 and 30 cm depth, both variables affected or interacted to affect community composition (Figure 7).

When comparing basalt addition to the UTC treatment (no MP1, no basalt), there were 71 OTUs that were differentially abundant. When comparing MP1 addition to the UTC, we identified 86 differentially abundant OTUs. When comparing MP1 plus basalt addition (MP1 + B) to the UTC treatment, we identified 76 differentially abundant OTUs (Table 1). Differentially abundant OTUs were from a total of 17 phyla (Table S3). Phyla that contained multiple differentially abundant OTUs generally contained some OTUs that increased in relative abundance in a given treatment and some that decreased in relative abundance with the same treatment. We also identified some OTUs that increased in relative abundance with the MP1 treatment and decreased in relative abundance with the basalt treatment and vice versa. For example, OTUs from Burkholderia and Gammaproteobacteria generally increased in MP1‐treated soils (MP1) and decreased in basalt‐amended soils (B), whereas Alphaproteobacteria OTUs decreased in MP1‐treated soils and increased in basalt‐amended soils. Mesocosms where MP1 was added with basalt treatment showed the same pattern as the MP1 treatment.

**TABLE 1:** Differential abundance unique and overlapping OTUs between the three comparisons done (Basalt, MP1, and MP1 + Basalt).

UTC vs. Basalt	UTC vs. MP1	UTC vs. MP1 + Basalt	OTUs (count)	% of total
✓			45	23.7
	✓		59	31.1
		✓	52	27.4
✓	✓		10	5.3
✓		✓	7	3.7
	✓	✓	8	4.2
✓	✓	✓	9	4.7

There were very few 16S rRNA gene reads recovered from amplicon sequencing of the four pure basalt samples (i.e., the basalt itself, rather than a mix of soil and basalt; mean = 119 ± 56 SD pre‐filtering; mean = 12 ± 13 SD post‐filtering). There were a total of 20 taxa identified on the basalt, and none of these matched OTUs that were differentially abundant in the basalt‐amended treatment. This suggests that changes in soil microbial communities associated with the basalt additions are due to indirect effects of the basalt on soil bacterial communities rather than direct additions of microbes associated with the basalt itself.

## 4 Discussion

### 4.1 Soil Chemistry

#### 4.1.1 Impact of Basalt Amendment on Soil Chemistry

Basalt amendment significantly affected soil and leachate chemistry, leading to increases in pH and export of base cations and inorganic carbon, compared with the no basalt mesocosms. The greater export of base cations and DIC in leachate from the basalt‐amended mesocosms reflects increases in chemical weathering, compared with unamended soils. These findings are consistent with prior studies (e.g., te Pas et al. 2023; Vienne et al. 2022; Conceição et al. 2022; Kelland et al. 2020) and support the prevailing assumption that additions of crushed silicate feedstocks can increase soil pH.

Depth‐dependent increases in CEC in the basalt‐amended soils (with and without MP1) suggest the formation of reactive secondary minerals, particularly in the deeper depth increments where the concentration of dissolved weathering products is more likely to approach saturation with respect to precipitating minerals. Accumulation of secondary weathering byproducts could reduce or delay the effects of enhanced weathering by scavenging cations (Steinwidder et al. 2025; Vienne et al. 2025). Conversely, accumulation of reactive minerals could increase the potential for forming mineral‐associated organic matter, which is considered a more stable form of organic carbon (Heckman et al. 2018; Kleber et al. 2015; Koegel‐Knabner et al. 2008; Kalbitz et al. 2005). However, in the present study, significant increases in CEC did not coincide with increased SOC (more details below). The overall impact of CEC increases may be greater in natural soils, where longer flowpaths and time provide greater opportunity for secondary mineral accumulation.

Multivariate analyses of the complete datasets for soil and soil leachate indicate that the addition of basalt is the primary driver of data clustering (Figure 4). This finding is consistent with the metagenomic analyses, which also indicate that basalt amendment exerts a stronger influence than MP1 on shifts in soil properties and microbial community composition (Figure 7 and Figure S4). It remains an open question whether this result would hold in more alkaline soils that are optimal for MP1‐driven weathering increases, as demonstrated by Timmermann et al. (2025).

#### 4.1.2 Impact of Microbial Amendment on Soil Chemistry

There was limited evidence for microbial enhancement to weathering as a result of the MP1 treatment, with or without basalt amendment. First, there were some significant differences between the microbial treatment (MP1) and the control (UTC), including higher soil carbonate alkalinity (in both basalt‐amended and unamended soil) and higher soil and leachate pH (only in unamended soils). Additionally, mean values for alkalinity and total cation fluxes were consistently higher in MP1 treatments; however, these differences did not reach statistical significance. A post hoc power analysis suggests that confirming these biological effect sizes, which are geologically meaningful but smaller than the primary mineralogical effects, would require replication levels (*N* > 25) that are challenging in complex mesocosm designs.

Given the results of Timmermann et al. (2025) and Niron et al. (2024), which each showed increases in weathering in the presence of *B. subtilis* , the absence of statistically significant changes in cation export within the study timeframe, in addition to the replication level, may be attributed to two primary factors. First, the starting pH of the soil used in this study is on the low end of the range identified as optimal for the methodology described in Timmermann et al. (2025), where the CO_2_ removal is calculated based on the concentration of SIC as part of a measure and re‐measure protocol. We hypothesize that the contribution of MP1 to silicate mineral weathering is dependent on the soil environment pH. MP1 can facilitate weathering via two mechanisms: concentrating acidity at the mineral surface and simultaneously favoring the precipitation of carbonate minerals (Timmermann et al. 2025). Importantly, the latter acts as a critical sink for weathering products; without it, the extent of silicate mineral weathering can be limited by reductions in reaction affinity as the system approaches equilibrium with regard to the weathering silicate minerals (e.g., Maher et al. 2009; Opolot and Finke 2015). Because the stability of biogenic carbonates is governed by soil pH, and the soil in this study was slightly acidic (pH 6.6), we observed limited evidence for increased SIC in any treatment. Consequently, the lack of a stable carbonate sink likely constrained the overall weathering efficacy. Although soil pH increased with the MP1 and/or basalt amendments, more time may be required to detect significant effects. For example, significant changes resulting from weathering typically occur over multi‐year timescales (e.g., Dupla et al. 2024) and in both mesocosm and field experiments, the proportion of applied basalt feedstock weathered varies over time, for example, from 6% after 99 days (Vienne et al. 2022), to 10% after 235 days (Reershemius et al. 2023), to 16% ± 6% after 4 years (Beerling et al. 2024).

In addition to a longer experimental period, more detailed characterization of leachate and soil geochemistry, combined with reactive transport modeling (e.g., Kanzaki et al. 2025) are needed to identify the type of secondary minerals that may be forming in this experimental system. For example, Vienne et al. (2025) investigated basalt weathering rates in corn cultivation mesocosms by tracking cation dynamics across four distinct soil pools (i.e., exchangeable, carbonate, reducible, and oxidizable pools) to gain a more comprehensive understanding of weathering processes. Niron et al. (2024) employed a similar sequential extraction approach and found that the majority of cations, such as Ca^2+^ and Mg^2+^, released from basalt dissolution were retained in the soil in reducible (e.g., secondary minerals) and oxidizable (e.g., organic matter) soil pools, rather than exported. Formation of secondary minerals, including (hydr)oxides and clays, is a potentially important limit on CDR (Vienne et al. 2025; te Pas et al. 2025). For example, in a 15‐month mesocosm study including basalt and steel slag, Steinwidder et al. (2025) determined that the realized CDR remained negligibly low as the majority of base cations were retained in soil pools, such as secondary (hydr)oxides.

The increases in CEC associated with basalt amendment (Figure 1) observed here suggest that secondary mineral formation may be a factor limiting the effectiveness of CDR in this study. Given this evidence of secondary mineral formation, despite the relatively short time frame of this study, a more detailed characterization of cation distribution across different geochemical pools could provide valuable insights into how secondary phase accumulation may influence CDR calculations based on cation accounting.

#### 4.1.3 Soil Inorganic Carbon as a Proxy for Weathering

There was limited evidence for increases in SIC in any combination of treatments (data not shown). Despite significantly higher soil alkalinity as estimated from saturated paste titrations in the MP1‐amended soils (Figure 1), no significant changes in total SIC were observed. It is possible that differences in SIC were not quantifiable due to the detection limit of the SIC method used here. However, the lack of detectable SIC despite measurable DIC fluxes (Figure 3) suggests that the aqueous transport regime, driven by the volume of water used in column leaching, may have inhibited accumulation of carbonates.

Other short‐term mesocosm studies also failed to detect significant changes in SIC involving basalt‐amended soils (Vienne et al. 2022; Kelland et al. 2020). For example, modeling by Vienne et al. (2022) estimated that approximately 5 years would be required to accumulate as little as 0.05% SIC in a mesocosm setting. Overall, these results highlight the need for longer‐term experiments or more sensitive evaluation methods. For instance, an approach such as multi‐pool carbon accounting (e.g., Sokol et al. 2024) could help to capture subtle changes in early‐stage C dynamics and improve assessment of the carbon sequestration potential of basalt amendments under field‐relevant conditions.

#### 4.1.4 Soil Organic Carbon

Several recent studies have shown that EW feedstock amendments can impact SOC storage (Steinwidder et al. 2025; Boito et al. 2025; Sohng et al. 2025; Sokol et al. 2024; Buss et al. 2024; te Pas et al. 2023). As SIC was near the detection limit of our method (Yip et al. 2025) for all soils in this study, the observed treatment effects on total carbon (Figure 1) are driven by differences in SOC. Interestingly, over the course of the experiment, the addition of basalt coincided with a negative effect on total carbon stocks in the top two depth increments. This observation is consistent with work by te Pas et al. (2023) and Steinwidder et al. (2025) that showed increases in net CO_2_ emissions from basalt‐amended soils. Those authors speculated that increases in CO_2_ efflux resulted from an enhanced decomposition driven by elevated soil pH. However, in this study, we observed no comparable reduction in SOC when the soil was amended with both basalt and the MP1 microbe, despite comparable increases in pH of that treatment.

The priming effect (Kuzyakov et al. 2000; Fang et al. 2023) is perhaps a more plausible mechanism through which CO_2_ efflux could be increased as a result of basalt amendments. Fang et al. (2023) documented that rock‐derived nutrient release by mineral weathering is closely linked to priming of soil organic matter decomposition via ‘triggered microbial activity,’ and that the congruence of weathering (i.e., the degree to which minerals dissolve completely versus forming secondary solid phases) modulates the accessibility of organic matter to decomposers and thus the priming effect. In the present study, a possible basalt‐driven priming effect on SOC decomposition in the upper two soil increments appeared to be suppressed in the presence of MP1, where reductions in carbon losses were minimized (Figure 1). Niron et al. (2024) reported a similar finding when comparing changes in CO_2_ efflux between basalt‐amended soils with or without the addition of *B. subtilis* . In the context of Fang et al. (2023), differences in the products of weathering in the presence of MP1 (or related microbes) versus when basalt is added alone may modulate SOC losses due to priming. This could include formation of mineral‐associated organic matter (MAOM), which has been linked to enhanced microbial metabolism in enhanced weathering (Buss et al. 2024) or increased soil aggregation driven by microbial biofilm formation (Krause et al. 2019).

#### 4.1.5 Potential Synergies of Basalt and Microbial Amendments

Interestingly, the strongest results in terms of weathering indicators were observed with the combination of basalt with MP1. We hypothesize that the increase in soil pH evidenced in the basalt‐amended soils (0.4 points, reaching an average pH of 7.3 in UTC + Basalt soils and 7.4 in MP1 + Basalt soils) could have resulted from MP1's carbonic anhydrases (CAs) activity. Microbially produced CAs have been postulated as a potential mechanism of action behind soil silicate mineral weathering (Xiao et al. 2015; Vicca et al. 2022; Timmermann et al. 2025).

The co‐application of basalt and MP1 presents a promising avenue for future research in real agricultural systems, particularly in soils with slightly acidic to neutral conditions. In such environments, the synergistic interaction between these amendments becomes more relevant: crushed basalt application can effectively raise soil pH toward the optimal range for MCM. While the mechanisms underlying the MCM approach are still under study, one hypothesis is the action of CAs which have an optimal activity at neutral pH (Sauze et al. 2018), thereby maximizing the functional potential of the microbial amendment. However, this co‐application strategy may offer diminishing returns in neutral to alkaline soils (pH ≥ 7), where the pH‐buffering capacity of basalt becomes less critical and the MP1‐mediated MCM has significant CDR potential without basalt amendments (e.g., Timmermann et al. 2025).

### 4.2 Soil Microbial Community

#### 4.2.1 Establishment and Persistence of MP1

While there have been many research trials on microbial soil amendments, many fail to track the success of the amendment over time. Microbial amendments may not establish in the community when there is already a diverse and established community. In this mesocosm experiment, we inoculated MP1 as a seed treatment and after 91 days of experiment, we detected MP1 in the bulk soil of all the soil columns that received the seeds treated with MP1. The two detection methods employed—metagenomics and qPCR—were in agreement. These findings suggest that the inoculation was successful. While we do not know if MP1 was actively growing in soil, prior research suggests that *B. subtilis* soil amendments are actively maintained. For example, in a field setting, an antibiotic‐resistant *B. subtilis* strain survived at least 100 days after inoculation in the rhizospheres of wheat plants (Van Elsas et al. 1986). The population was maintained around 10^3^ CFU·g^−1^ of soil throughout the experiment after inoculation with 10^7^ CFU·g^−1^ of soil. The population of cells at any time point ranged from all vegetative to all spores, suggesting growth and maintenance of the bacterial population throughout the experiment. The similar level of maintenance of MP1 in our experiment over a comparable time period offers the possibility that MP1 may also be actively maintained in the rhizosphere. In future studies, we aim to implement a dual approach combining quantitative PCR with viable cell counts to quantify MP1 abundance and viability specifically in root and rhizosphere samples. This approach will allow us to distinguish between total and metabolically active populations, thereby providing a more comprehensive understanding of MP1 persistence at its primary colonization sites (i.e., the plant roots and surrounding rhizosphere soil). We hypothesize that MP1 cell concentrations in these compartments will exceed those observed in the bulk soil.

#### 4.2.2 Impact of Basalt and MP1 Treatments

After soil depth, basalt was the second most influential factor affecting soil community composition, significantly impacting community composition at all three depth increments (Figure 7). We postulate that this is an indirect effect driven by the significant soil pH increase detected in the basalt‐amended soils (Figure 1), which aligns with foundational continental‐scale studies by Fierer and Jackson (2006), Lauber et al. (2009) and Bahram et al. (2018). These studies demonstrated that soil pH is the primary environmental factor controlling the diversity, richness, and overall structure of soil bacterial communities across diverse ecosystems. Still, the effect size of basalt was low, suggesting that basalt amendments are associated with significant yet subtle effects on the pre‐existing soil microbial community.

Notably, in the upper 10 cm of soil, where MP1 was detected, the presence of MP1 had no significant effect on soil community composition, in contrast to basalt addition, which induced significant compositional changes. This suggests that as a microbial soil amendment, MP1 adds benefit to the soil without changing the overall microbiome composition of the soil community. This is important as it suggests that inoculation with MP1 is unlikely to have strong effects on pre‐existing soil bacterial communities.

Conversely, addition of both MP1 and basalt changed the community at the lower column depths, beyond where MP1 was identified by sequencing and qPCR, possibly due to alterations in soil chemistry that affected the lower layers. For example, this could be due to downward transport of weathering products from the top layers. Accordingly, we observed increases in CEC (Figure 1), particularly with basalt treatment, in the deeper soil layers. It is also possible that these changes in the microbial community could be driven by other depth‐associated variables that we did not measure such as soil moisture or oxygen content. Finally, the MP1 treatment may have changed the root architecture or secretion in some way that led to a plant‐associated change in microbial community structure at lower depths.

While changes in microbial community composition were observed in the middle layer of the column, where MP1 was not detected, no significant shifts were found in the top layer, where MP1 persisted. This suggests that, unlike many microbial inoculants (Mawarda et al. 2020), MP1 may exert only a limited direct impact on the pre‐existing soil microbial community into which it is introduced. Nevertheless, within the subtle effect on the pre‐existing soil microbial community observed with MP1 treatment, there was a selective enrichment of taxa within the bacterial orders Burkholderiales and Bacillales, suggesting that this microbial amendment may serve as a molecular signal that recruits specific microbial taxa with enhanced weathering capabilities, as members of these groups have been documented to have mineral‐weathering potential (Uroz et al. 2007, 2009, 2022; Wang et al. 2014, 2018; Zhang et al. 2015). The preferential proliferation of these taxonomic groups following MP1 application suggests that MP1 may orchestrate a targeted recruitment of microbial partners capable of enhancing silicate mineral dissolution, potentially accelerating the weathering of cation‐bearing rocks and the subsequent release of essential nutrients. Studying the potential synergistic effects among MP1 and other mineral‐weathering taxa is an avenue for future research.

## 5 Conclusion

In basalt‐amended soils, MP1 co‐application was associated with higher total carbon concentrations in the upper soil profile compared to the basalt‐only treatment, while soil inorganic carbon remained near detection limits throughout the study period. The observed increases in total carbon associated with MP1 were primarily attributed to organic carbon, potentially reflecting a mitigation or offset of the decomposition priming effect induced by basalt amendments. After 91 days, MP1 remained detectable in treated soils, demonstrating its capacity for sustained persistence in the rhizosphere environment where CDR processes mediated by this microbial amendment occur. Multivariate analyses of both soil geochemical properties and microbial community composition consistently differentiated basalt‐amended from unamended soils, with MP1 effects being secondary and depth‐dependent. Microbial community shifts were driven primarily by soil depth and the presence of basalt, with minimal compositional changes occurring in the surface layer where MP1 persisted. The basalt‐induced community restructuring appeared to result from indirect geochemical effects, most likely mediated by significant increases in soil pH.

These findings indicate that co‐deploying basalt amendments with MCM in slightly acidic soils represents a viable strategy for carbon dioxide removal: basalt provides robust and immediate geochemical responses, while MP1 establishes persistent rhizosphere colonization, moderately elevated soil alkalinity, and potentially mitigates short‐term SOC losses observed in basalt‐only treatments. Collectively, these complementary mechanisms offer a practical framework for integrating microbial and crushed silicate rock amendments in agricultural systems to accelerate silicate weathering processes while preserving pre‐existing microbiome integrity.

## Author Contributions

**Yun‐Ya Yang:** writing – review and editing, writing – original draft, investigation, formal analysis, supervision, methodology, conceptualization, data curation. **Clifton P. Bueno de Mesquita:** writing – review and editing, writing – original draft, formal analysis, visualization, investigation. **Corey R. Lawrence:** writing – review and editing, writing – original draft, formal analysis, data curation, visualization. **Philip D. Weyman:** writing – review and editing, writing – original draft, investigation, project administration. **Daniel Dores:** writing – review and editing, investigation. **Tania Timmermann:** writing – review and editing, writing – original draft, supervision, project administration, formal analysis, methodology, conceptualization, investigation. **Noah Fierer:** writing – review and editing, resources, supervision. **Gonzalo A. Fuenzalida‐Meriz:** writing – review and editing, funding acquisition, supervision, resources, conceptualization.

## Conflicts of Interest

The authors declare potential conflicts of interest as follows: Y.‐Y.Y., C.R.L, P.D.W., D.D., T.T., and G.A.F.‐M. are employed by Andes Ag Inc., the company that funded this study. The author N.F. is a compensated member of the scientific advisory board of Andes Ag Inc.

## Supporting information


**Data S1:** gcb70705‐sup‐0001‐supinfo.pdf.

**Table S3:** Excel file comprising the differential abundance analysis at phyla level. ANCOMBC2 treatment vs. control (UTC). Basalt vs. UTC: 71 OTUs, MP1 vs. UTC: 86 OTUs, MP1 + Basalt vs. UTC: 76 OTUs. LFC = log fold change.

## Data Availability

The soil and leachate data that support the findings of this study are openly available in Zenodo at https://doi.org/10.5281/zenodo.17538160. The raw metagenomic sequences have been deposited to NCBI SRA under BioProject accession PRJNA1356872. Processed data and code for microbial analyses are publicly available via a GitHub repository released to Zenodo at https://doi.org/10.5281/zenodo.17526583.
