# Supplementary material for: Synergistic Effects of a Microbial Amendment and Crushed Basalt: Soil Geochemical and Microbial Responses
Source: Glob Chang Biol. 2026 Jan 17;32(1):e70705. doi: 10.1111/gcb.70705 (PMC12811823; doi:10.1111/gcb.70705)
Supplement: Supplementary file 1 — Data S1: gcb70705‐sup‐0001‐supinfo.pdf. [file GCB-32-e70705-s002.pdf]

Supplementary Information for "Synergistic Effects of a Microbial Amendment and Crushed Basalt: Soil Geochemical and Microbial Responses"

Yun-Ya Yang[1], Clifton P. Bueno de Mesquita[2,3], Corey R. Lawrence[1], Philip D. Weyman[1], Daniel Dores[1], Tania Timmermann[1], Noah Fierer[2,3], Gonzalo A. Fuenzalida-Meriz[1,]*

[1] Andes Ag, Inc., Alameda, California, USA

[2] Department of Ecology and Evolutionary Biology, University of Colorado Boulder, Boulder, Colorado, USA

[3] Cooperative Institute for Research in Environmental Science, University of Colorado Boulder, Boulder, Colorado, USA

[*] Corresponding Author: Gonzalo A. Fuenzalida-Meriz, gonzalofuenzalida@gmail.com

FIGURES AND TABLES

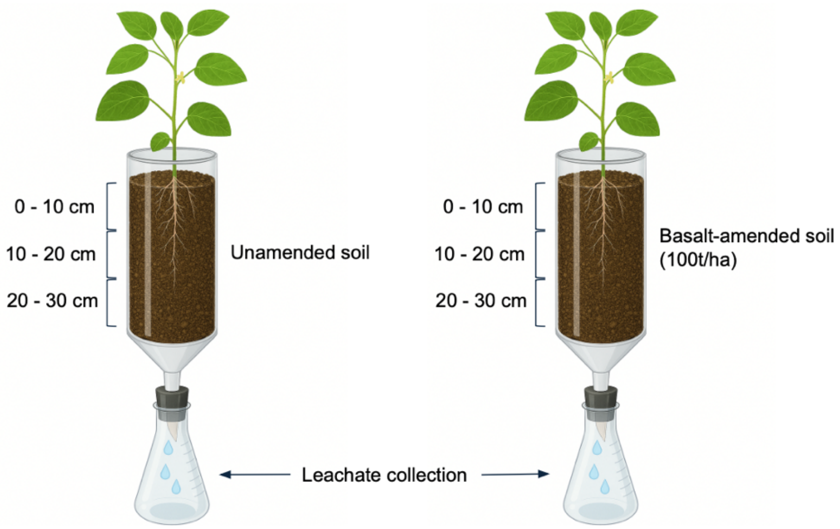

Treatment	MP1	Basalt
UTC (x 6 columns)	-	-
MP1 (x 6 columns)	+	-
B (x 6 columns)	-	+
MP1+B (x 6 columns)	+	+

Figure S1. Scheme of the mesocosm study design.

27

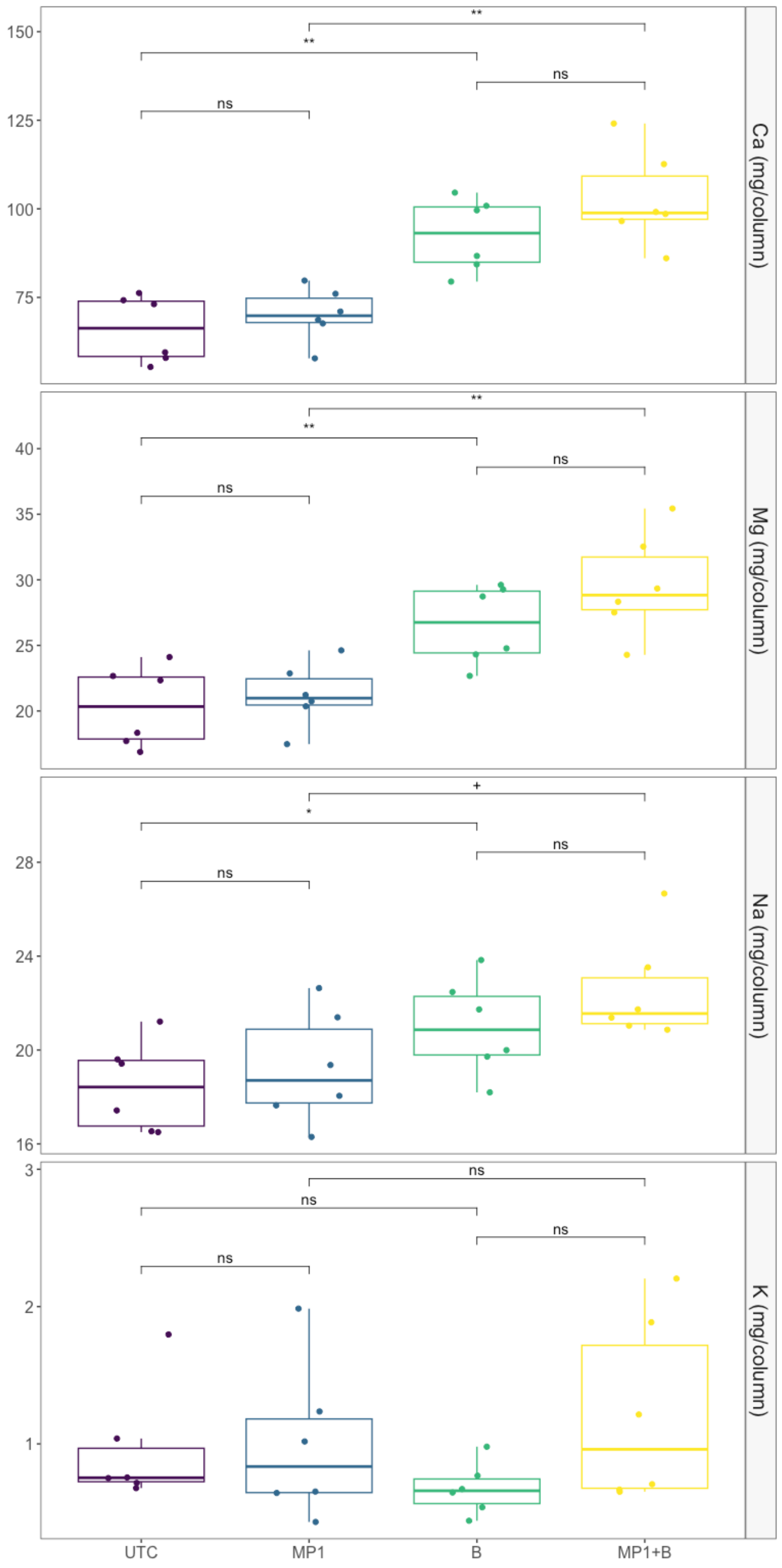

Ca (mg/column)
Mg (mg/column)
Na (mg/column)
K (mg/column)
UTC
MP1
B
MP1+B
ns
**
+
*
150
125
100
75
40
35
30
25
20
28
24
16
3
2
1

Figure S2. Total leachate fluxes of Ca^{2+}, Mg^{2+}, Na^{+}, and K^{+} fluxes summed over all wetting events. Groups are compared using the non-parametric Mann-Whitney U (Wilcoxon rank-sum) test. The number above the brackets indicates the *p*-value. Note that y-axes are scaled to the data range to clearly visualize the distribution and variability across treatments.

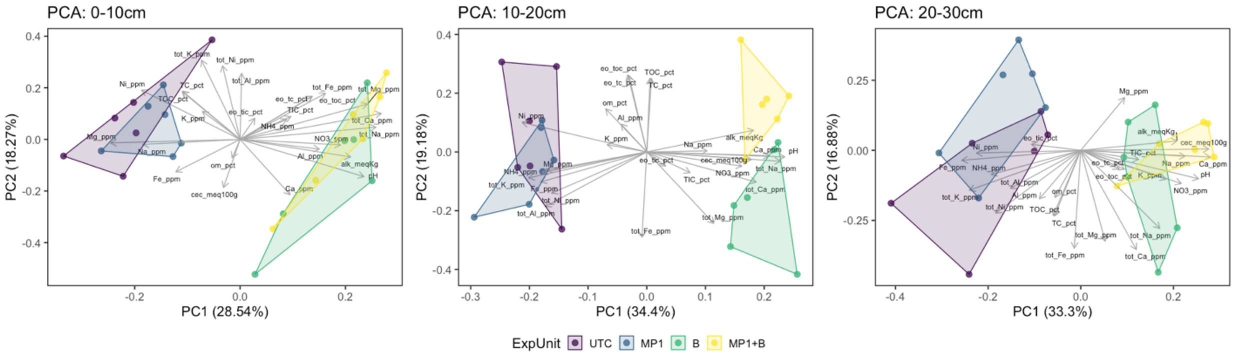

Figure S3. Principal component analysis (PCA) of soil data partitioned by column depth increments. Individual points represent replicated columns.

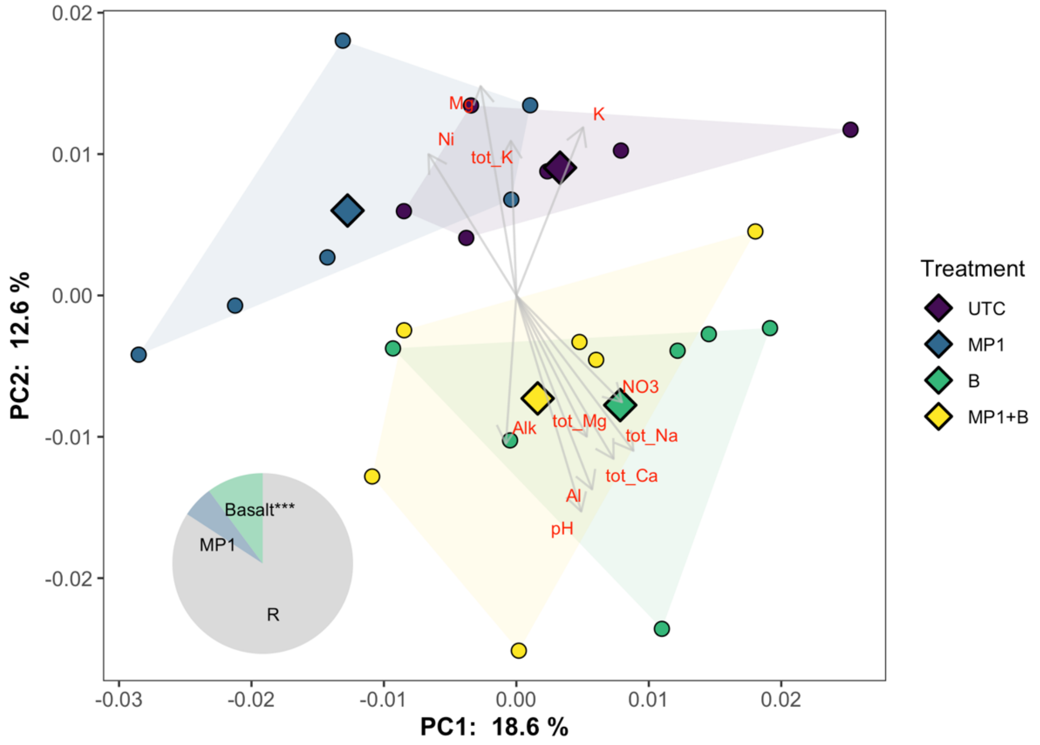

Figure S4. Principal coordinates analysis of Bray-Curtis dissimilarity at the phylum level for the 0-10 cm depth increment, with hulls showing the clustering by treatments, and vectors showing associations with soil variables (envfit, $p < 0.05$). Diamonds represent the treatment centroids. Pie inset shows the effect sizes (eta-sq) from ANOVA (*** $p < 0.001$).

Table S1. The mineral abundance of soil and basalt were measured via semi-quantitative XRD. The Soil+Basalt* values were calculated based on a mixture of 78 g of basalt in 2146 g of soil (dry weight).

Mineral	Soil (%)	Basalt (%)	Soil+Basalt* (%)
Quartz	50	9	49
K-Feldspar	16	0	15
Albite	14	0	14
Other Silicates	11	0	9
Pyroxene/Amphibole	9	28	10
Anorthite	<2	63	4

Table S2. Abundance of major oxides measured by XRF in the soil and the Soil+Basalt mixture. Loss-on-ignition (LOI) represents the mass of volatile compounds in the sample, including organic matter and structural water in clays. The basalt* values were calculated based on a mixture of 78 g of basalt in 2146 g of soil (dry weight).

Elemental Oxide	Soil (wt %)	Basalt* (wt %)	Soil+Basalt (wt %)
SiO_2	70.6	49.1	69.8
TiO_2	0.59	0.9	0.60
Al_2O_3	10.4	13.0	10.5
FeO	3.29	15.9	3.73
MnO	0.11	0.2	0.11

MgO	0.89	6.6	1.09
CaO	1.24	10.1	1.55
Na_2O	1.37	2.8	1.42
K_2O	1.95	0.5	1.90
P_2O_5	0.16	0.0	0.15
LOI	9.09	3.1	8.88

Table S3. Excel file comprising the differential abundance analysis at phyla level. ANCOMBC2 treatment vs. control (UTC). Basalt vs UTC: 71 OTUs, MP1 vs UTC: 86 OTUs, MP1+Basalt vs UTC: 76 OTUs. LFC = log fold change.
